# The metabolic conditioning of obesity: A review of the pathogenesis of obesity and the epigenetic pathways that “program” obesity from conception

**DOI:** 10.3389/fendo.2022.1032491

**Published:** 2022-10-18

**Authors:** Ananthi Rajamoorthi, Charles A. LeDuc, Vidhu V. Thaker

**Affiliations:** ^1^ Department of Pediatrics, Columbia University Medical Center, New York, NY, United States; ^2^ The Naomi Berrie Diabetes Center, Columbia University IRVING Medical Center, New York, NY, United States; ^3^ Vagelos College of Physicians and Surgeons, Columbia University, New York, NY, United States

**Keywords:** childhood obesity, metabolic programing, epigenetics, *in utero* environment, cardiometabolic risk, postnatal conditions

## Abstract

Understanding the developmental origins of health and disease is integral to overcome the global tide of obesity and its metabolic consequences, including atherosclerotic cardiovascular disease, type 2 diabetes, hyperlipidemia, and nonalcoholic fatty liver disease. The rising prevalence of obesity has been attributed, in part, to environmental factors including the globalization of the western diet and unhealthy lifestyle choices. In this review we argue that *how* and *when* such exposures come into play from conception significantly impact overall risk of obesity and later health outcomes. While the laws of thermodynamics dictate that obesity is caused by an imbalance between caloric intake and energy expenditure, the drivers of each of these may be laid down before the manifestation of the phenotype. We present evidence over the last half-century that suggests that the temporospatial evolution of obesity from intrauterine life and beyond is, in part, due to the conditioning of physiological processes at critical developmental periods that results in maladaptive responses to obesogenic exposures later in life. We begin the review by introducing studies that describe an association between perinatal factors and later risk of obesity. After a brief discussion of the pathogenesis of obesity, including the systemic regulation of appetite, adiposity, and basal metabolic rate, we delve into the mechanics of how intrauterine, postnatal and early childhood metabolic environments may contribute to adult obesity risk through the process of metabolic conditioning. Finally, we detail the specific epigenetic pathways identified both in preclinical and clinical studies that synergistically “program” obesity.

## Key Messages

1. Obesity is the result of a dynamic interplay between genetics, physiology, behavior, and environment, that accumulate over time from conception and can predispose to energy homeostasis perturbations throughout the lifespan.
2. Developmental plasticity allows for the conditioning or “programming” of obesity in response to the metabolic environments of intrauterine, postnatal, and early childhood, that may be responsible for resistance to weight loss efforts in later life.
3. The metabolic environments in the prenatal, postnatal, and early childhood periods, reflective of maternal nutrition, postnatal feeding, and early childhood adiposity, respectively, significantly contribute to adult health outcomes.
4. Emerging evidence suggests that *in utero*, postnatal and early childhood metabolic environments affect adult basal metabolic rate and, in turn, obesity risk through metabolic programming, that needs further exploration.
5. Altered maternal levels of metabolic substrates and hormones in obesity contribute to maternal insulin resistance, oxidative stress and inflammation, resulting in perturbations in glucose and lipid homeostasis and feeding circuitry in the perinatal period, contributing to long-term obesity risk.
6. The *in utero* environment of maternal obesity, and the concomitant short-term aberrations in energy homeostasis during the perinatal period, translate to long-term obesity risk through both central and peripheral epigenetic modifications that regulate energy homeostatic set points.
7. Epigenetic changes are not exclusive to the perinatal period; these changes may occur during childhood and beyond in response to behavioral and environmental factors.
8. Epigenetic pathways that contribute to the conditioning of obesity are highly dynamic and reversible. Currently known pathways include variations in methylation status of obesity-related genes, which occur in response to maternal, postnatal, and early childhood nutrition, and have been shown to be associated with differences in adiposity and body composition in infancy and beyond.

## Introduction

### Early life exposures “program” long-term obesity risk

#### The global impact of childhood obesity

Childhood obesity has become one of the most pervasive global health crises of this century. In 2020, 39 million children worldwide under the age of 5 years had overweight or obesity. The prevalence of overweight and obesity in children aged 5-19 years across genders quadrupled from 4% in 1975 to over 18% in 2016. Further, what was once considered to be an epidemic of the Western world, childhood obesity has infiltrated both low- and middle-income countries. Since 2000, there has been a 24% increase in the prevalence of overweight/obesity in children under 5 years in Africa, while 50% of all children under 5 years with overweight and obesity lived in Asia in 2019 (1).

Obesity impacts growth and development from infancy, and is associated with increased risk of body dissatisfaction, depressive symptoms and low self-esteem during childhood and adolescence (2, 3). Childhood obesity persists into adulthood and is associated with higher prevalence of cardiometabolic risks including atherosclerotic cardiovascular disease (ASCVD), type 2 diabetes (T2D), hyperlipidemia and nonalcoholic fatty liver disease (NAFLD) (4).

#### Systemic regulation of adiposity and metabolic rate

Many genes associated with body mass index (BMI) are expressed in the hypothalamus, which receives sensory inputs from peripheral organs including the gastrointestinal (GI) tract, liver, adipose tissue, pancreas, and skeletal muscle regarding the overall metabolic status of the organism, to regulate food intake, appetite, and body weight (5). The “gut-brain-axis” is critical for some of these inputs due to the presence of mechanosensors and chemoreceptors in the intestinal epithelial wall that sense both volume and nutrient content of ingested food. This information is communicated centrally *via* two mechanisms: vagal innervation and endocrine hormones. GI-derived hormones, such as glucagon like peptide-1 (GLP-1) and ghrelin, not only behave in a paracrine manner to affect the local absorption and metabolism of nutrients, but can also act centrally through signaling pathways to affect feeding behavior and whole-body energy homeostasis (6). Hormones produced by adipose tissue, pancreas, skeletal muscle, and liver also serve as sensory inputs for the hypothalamus, including leptin, cholecystokinin, peptide YY, insulin, fibroblast growth factor 21 (FGF21) and other “adipokines” and “myokines,” which regulate systemic lipid and carbohydrate homeostasis (7). The level of leptin, produced by adipose tissue, is directly proportional to its mass. Its functional roles during early stages of development are diverse (8). In adults, however, reduction and/or absence of leptin promotes food intake and decreases energy expenditure (9). Together with the corticolimbic system and hindbrain, the hypothalamus integrates peripherally derived inputs to regulate appetite and food intake.

In addition to food intake, energy expenditure (EE) is an important component of metabolic homeostasis and is largely a combination of basal metabolism, thermogenesis, and physical activity. While central neuronal networks play a role in the regulation of EE, the critical component of EE, basal metabolic rate (BMR), is primarily determined by fat-free mass. Fat-free or “lean” mass is composed of skeletal muscle, which together with other metabolically active organs including the liver, heart, brain and kidneys, contribute to BMR (10). Skeletal muscle is a major player for insulin mediated glucose disposal; and dysregulation of skeletal muscle metabolism can strongly influence glucose homeostasis and insulin sensitivity predisposing to obesity related metabolic diseases (11).

Based on the laws of thermodynamics, obesity is a consequence of an imbalance of food intake and energy expenditure. But *how* and *when* such perturbations take place, and in what circumstances and metabolic environments they are more likely to occur, plays a vital role in determining the overall risk of obesity which will be discussed in the following sections.

#### The role of “programming” in the pathogenesis and treatment of obesity

Obesity is the result of a dynamic interplay between genetics, physiology, behavior, and environment, that accumulate over time since conception and can predispose to perturbations in energy homeostasis throughout life. The most widely studied and accepted interventions for obesity are lifestyle modifications, including exercise and dietary changes. However, both the efficacy and sustainability of such interventions are questionable, as the majority of individuals with obesity are unable to maintain reduced weight despite effective weight loss (12).

Socioeconomic factors, including accessibility to resources, are important limitations to the overall success of lifestyle interventions. As obesity often affects populations of limited socioeconomic resources, lifestyle modifications are challenging to initiate and sustain in the populations that would most benefit from such interventions, often due to a lack of resources and low health literacy (13, 14).

Further, if obesity was simply driven by an imbalance between food intake and expenditure at a single time point, lifestyle interventions would result in more positive and consistent outcomes. However, despite many public health measures promoting healthy lifestyle habits and health care spending on the screening and prevention of obesity and metabolic syndrome, obesity related ASCVD remains the leading cause of morbidity and mortality in the US and worldwide (15, 16).

**Figure 1:**
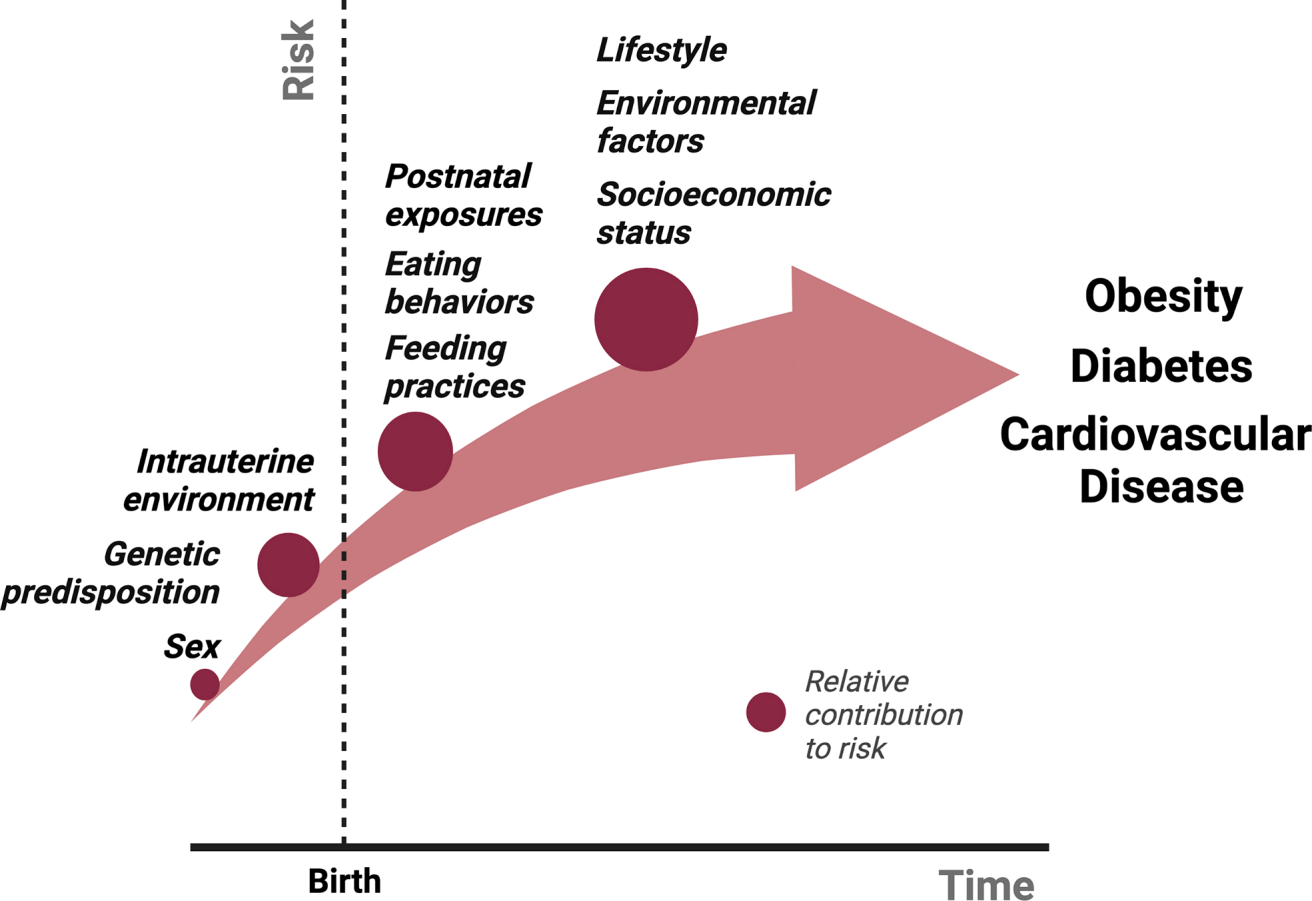
The relative contributions of various exposures to the cumulative risk for obesity over time from conception. Several factors contribute to the pathogenesis of obesity. We propose that intrauterine and postnatal exposures make relatively large contributions to the cumulative risk for obesity, in addition to the genetic predisposition. Studies described in this review have shown that postnatal exposures could potentially reverse the pathological events of intrauterine life in the setting of either maternal undernutrition or obesity, suggesting a more significant role for the postnatal environment in the “programming” obesity. We hypothesize that obesity risk acquired beyond early life, primarily in adolescence and adulthood through poor eating and lifestyle habits potentiated by socioeconomic status and other environmental factors, make an even greater contribution to obesity risk due to the sheer amount of time and opportunities available to accrue risk, although this is largely dependent on the individual.

There are several physiological mechanisms that explain why sustained weight loss through lifestyle modifications remains difficult (7, 17). Caloric restriction, both in short- and long-term, reduces 24-hour EE (18–20). In addition, several neuroendocrine hormones fluctuate in response to either caloric restriction or acute weight loss, including reductions in leptin and cholecystokinin and an increase in ghrelin, which ultimately function to increase appetite and promote weight regain. Sumithran et al. found that these hormonal fluctuations and increased appetite following 10% weight loss over 8 weeks (and subsequent weight stabilization), persist at 1 year (7). What causes these acute and chronic fluctuations in neuroendocrine signaling that ultimately thwart intervention and promote obesity? While genetic predisposition, socioeconomic factors, lifestyle habits and environmental exposures all contribute to the risk of obesity, the metabolic environment during early development lays the critical foundation for the conditioning of obesity and long-term ASCVD risk (**Figure 1**).

Studies discussed in the following sections show a clear association between perinatal and childhood metabolic exposures including maternal under- and over-nutrition, maternal diabetes, breastfeeding, early infancy weight gain, and later health outcomes, including obesity and T2D. We hypothesize that developmental plasticity allows for the conditioning or “programming” of obesity in response to the metabolic environments of intrauterine, postnatal, and early childhood life, that play an important role in the resistance to weight loss efforts in later life. Further, if obesity is, in part, the result of conditioning or “programming” of certain physiologic processes established during critical developmental periods of early life, then lifestyle interventions initiated during adulthood will be insufficient to revert the initial “code” (21, 22). Addressing the risk for obesity in mothers prior to conception or during these critical developmental periods will likely produce favorable long-term outcomes.

#### The “developmental origins of health and disease” hypothesis and defining the phenomenon of metabolic programming

Metabolic programming (also called developmental programming) refers to the contribution of environmental exposures and nutrient imbalance from conception to early postnatal life – the critical times of organ development and maturation – to the risk of obesity and metabolic syndrome in adulthood, even after the absence or reversal of such exposures (17, 21–23). This is a more focused topic within a larger field known as the developmental origins of health and disease, or simply called “DOHaD.” Barker and other experts coined this concept as the “fetal origins of disease” in the 1990s (17, 23–26). Both terms refer to a similar phenomenon in which exposures during critical developmental periods affect long-term health outcomes.

Many experts argue that the term “programming” is not accurate or reflective of the reversible and dynamic nature of these physiologic yet nonadaptive responses and prefer the term “conditioning” instead (17). Throughout this review, accepting both the limitations of the word “programming” as well as its prevalent use in scientific literature, we will interchangeably use these terms referring to the same phenomenon.

## Epidemiological studies supporting the role of metabolic programming of obesity

### Risk factors for obesity acquired during different periods of development

#### Prenatal period

The metabolic environments in prenatal, postnatal, and early childhood, which are, in turn, reflective of maternal nutrition, postnatal feeding and early childhood adiposity, respectively, contribute to adult health outcomes. One of the earliest insights into the effects of the *in utero* environment on risk of obesity is from the evaluation of the Dutch “Hunger Winter:” the tragic food restriction imposed upon much of the Northern Holland population by Germany during World War II (27). Over a 6-month period (October 1944 to April 1945), caloric intake was markedly decreased from 1500 to 500 kcal, including that for pregnant women. Exposure to maternal undernutrition during the first or second trimesters increased the prevalence of obesity and cardiometabolic risk in the offspring at 18 years of age, when compared to those exposed in the third trimester.

There is significant controversy on the association of birth weight and later obesity risk. In a series of studies conducted since 1989, Barker et al. showed that individuals from the UK with low birth weight had higher risk of coronary heart disease and other risk factors associated with metabolic syndrome including T2D and elevated blood pressure in adulthood (23–26). Based on these studies, Barker proposed that chronic, degenerative conditions of adulthood, including heart disease and T2D, may be triggered by malnutrition during the *in utero* period (fetal “programming”), remain latent for many years, and manifest later in life. Deng et al. found that children from Guangzhong, China with high birth weight had higher odds of overweight/obesity compared to infants with normal birth weight (Odds ratio [OR] 2.42, 95% confidence interval (CI): 1.56; 3.76) (28). Large scale studies have confirmed a U-shaped relationship between birth weight and long-term outcomes, with both higher and lower levels associated with increased risk of different magnitude (29, 30). Further, there is an additive interaction of high birth weight and insufficient physical activity, such that children with high birth weight and lack of physical activity were 3.75 times more likely of being overweight or obese at age 7-9 years compared to those with normal birth weight and sufficient activity (OR 3.75, 95% CI: 2.06; 6.83) (28).

But why such discrepancies? Birth weight, although can be considered a marker of fetal health, is not a holistic reflection of the *dynamics* of intrauterine metabolic environment over time. As shown in the Dutch famine study, the gestational age during which maternal caloric restriction occurs may affect later obesity risk differentially, suggesting the presence of critical developmental windows. Further, there are several paths that can lead to a low birth weight independent of maternal body composition, food/caloric intake, or nutritional status. Mild prematurity, as well as intrauterine growth restriction secondary to uteroplacental insufficiency in the setting of chronic or gestational hypertension, smoking exposure, et cetera may also contribute to low birth weight, although studies in the UK, USA and Australia have shown that the associations between low birth weight and cardiovascular disease risk are not the result of such confounding variables (31–34).

More importantly though, while Barker’s hypothesis on the effects of intrauterine life on later health outcomes has been confirmed, the “fetal origins of disease” hypothesis does not consider the metabolic environment in the postnatal period, that is also known to affect future obesity risk. Indeed, considering the exponential rise in the prevalence of ASCVD since Barker’s studies, individuals with birth weight across the spectrum are likely to be impacted.

Other prenatal factors such as maternal diabetes and obesity, have been shown to contribute to obesity risk in children both during infancy and later in life. A large population-based comprehensive cohort study evaluated the association between diabetes in the mother (diabetes with insulin treatment, non-insulin treated diabetes and gestational diabetes), stratified by maternal prepregnancy BMI, and large for gestational age (LGA) at birth (35). Pregnant mothers with increasing BMI in the absence of diabetes had increasing odds of having an LGA offspring (36, 37). The adjusted OR for mothers with prepregnancy BMI of 25-29, 30-34 and ≥ 35 having LGA infants were 1.91, 2.45 and 3.38, respectively (95% CI: 1.83-2.00; 2.29-2.62; 3.08-3.71). Risk for LGA in offspring of mothers with obesity doubled in the presence of gestational diabetes and roughly tripled with T2D. Mothers who had insulin-treated diabetes had the highest risk of having LGA offspring regardless of BMI (35).

The Hyperglycemia and Adverse Pregnancy Outcome (HAPO) Study, designed to assess the impact of hyperglycemia on short- and long-term outcomes on the offspring, showed higher frequency of birth weight over 90^th^ percentile, primary Caesarean section, and cord-blood serum C-peptide over 90^th^ percentile (a marker of fetal hyperinsulinemia) with increasing hyperglycemia while adjusting for maternal prepregnancy BMI (38, 39). Further, children of mothers with hyperglycemia showed higher levels of obesity, glucose intolerance and insulin resistance at 10-14 years of age (40, 41). These data suggest that maternal hyperglycemia, even if not clinically considered gestational diabetes, is an important risk factor for perturbations in energy homeostasis and obesity in children, independent from maternal hyperinsulinemia, insulin resistance or obesity, perhaps driven by a hyperglycemic *in utero* environment.

The 1996 National Longitudinal Survey of Youth, Child and Young Adult in the US showed that children at 2-14 years of age born to mothers with obesity prior to pregnancy were approximately four times more likely of being obese (95% CI: 2.6; 6.4, P < 0.001) (42). Additionally, a meta-analysis of data acquired from birth cohort studies from Europe, North America and Australia showed that higher maternal prepregnancy BMI and gestational weight gain were associated with higher risk of childhood overweight/obesity, most significantly in late childhood (10-18 years) (43).

### Postnatal period

In the postnatal period, breastfeeding is found to have a protective effect against obesity (44). In a systematic review, Horta et al. showed that children (age 1-9 years), adolescents (age 10-19 years) and adults (≥ 20 years) who were breastfed as infants, have a reduction in the prevalence of overweight or obesity by 26% (95% CI: 21%; 32%), 37% (95% CI: 27%; 46%) and 12% (95% CI: 6%; 18%) respectively (45). The protective effect was more substantial in youth compared to adults, likely due to the accumulation of other environmental factors that may dilute the impact of breastfeeding over time. Further, while most studies were from high-income settings, the effect was consistent across income classifications. Breastfeeding during infancy is also associated with reduced odds of T2D in subjects aged 10-19 years [pooled OR: 0.46 (95% CI: 0.33; 0.66)]. However, this effect was not statistically significant in subjects older than 20 years of age, re-emphasizing the impact of other contributors in adulthood (45).

### Early childhood

Weight gain during the first two years of life, is often used as a surrogate marker for overall metabolic status and has been shown to affect persistent risk for obesity (46, 47). Rapid weight gain during early infancy, especially during the first 4-6 months of life, is associated with higher risk of obesity both in childhood (at 6-8 years of age) and in adulthood (at 20 years). Eid et al. showed that the prevalence of obesity was greater in infants with rapid weight gain (9.4%) in the first 6 months of life compared to those with slower weight gain (1.9%) as early as 1970 (48). In 2002, in a large-scale prospective cohort study, Stettler et al. demonstrated a 38% (95% CI: 32%-44%) increased risk of overweight status in children aged 7 years for each 100 gram per month increase in weight gain during the first 4 months of life, independent of birth weight (49). Over two dozen studies have since shown a similar association between rapid early childhood weight gain and later obesity risk (50–54).

Accelerated weight gain during infancy also increases the long-term risk of insulin resistance (55). Singhal et al. measured 32-33 split proinsulin, a serum marker of insulin resistance, in adolescent populations (age 13-16 years) with a history of prematurity who received either nutrient-enriched or lower-nutrient diet during infancy. Premature infants fed nutrient-enriched formula had greater levels of fasting 32-33 split proinsulin (7.2 pmol/L, 95% CI 6.4–8.1) in adolescence compared to those fed lower-nutrient diet (5.9 pmol/L, 95% CI 5.2–6.4), with a mean difference of 20.6% (p = 0.01). Furthermore, fasting 32-33 split proinsulin concentration was associated with greater weight gain in the first two weeks of life (13.2% [5.4–20.9] change per 100 g weight increase; p = 0.001). These data indicate that relative undernutrition during the postnatal period in premature infants may be protective against development of insulin resistance later in life.

The UK-based Avon longitudinal study of parents and children (ALSPAC), found a significant association between weight gain in infancy and seven other independently associated factors with the risk of obesity in 7-year-old children: birth weight, parental obesity, sleep duration, television viewing, size in early life, catch-up growth and early adiposity or BMI rebound (before 43 months) (56, 57). Children in the highest quartile for weight at age 8 months and 18 months were more likely to have obesity at age 7 years with an OR of 3.13 (95% CI: 1.43; 6.85, p = 0.004) and 2.65 (95% CI: 1.25; 5.59, p = 0.011), respectively. Children with a history of rapid catch-up growth between 0-2 years were approximately 2.6 times more likely to be obese at 7 years of age (OR 2.60, 95% CI: 1.09;6.16, p = 0.002). Adiposity rebound, which corresponds to the second rise in BMI that typically occurs between 5-7 years of age, was associated with later obesity risk. Specifically, early adiposity rebound (by 61 months) and very early adiposity rebound (by 43 months) were both associated with risk of obesity at 7 years, with ORs of 2.01 (95% CI: 0.81; 5.20, p < 0.001) and 15.00 (95% CI: 5.32; 42.30, p < 0.001) respectively.

Collectively, these studies describe associations between *in utero*, postnatal, and early infancy factors that affect one’s metabolic environment, predisposing not only to childhood obesity but also metabolic syndrome. Before taking a deeper dive into why such associations may exist, we will first define obesity and briefly summarize the factors that affect whole-body adiposity and metabolic rate, both of which contribute to the pathogenesis of obesity.

### A word on BMI and other measures of obesity

In studies discussed throughout this review, various markers of obesity were used as proxy measures of fat accumulation. BMI (weight [in kg]/height^2^ [in m]) is often used as a marker of excessive fat accumulation and is the most common measure of obesity in epidemiological studies over the age of 2 years. The World Health Organization (WHO) defines obesity in adults as BMI ≥ 30 kg/m^2^. In children under 5 years, BMI-for-age, and weight-for-height measurements according to specific growth standards are frequently used (47). The WHO defines obesity as weight-for-height ≥ 3 standard deviations above the median WHO Child Growth standards, while the Centers for Disease Control (CDC) defines childhood obesity as BMI ≥ 95^th^ percentile for sex and age as per the CDC 2000 growth charts.

There are several limitations to the use of BMI as a marker of fat accumulation. The calculation of BMI includes both body weight and height. However, body weight reflects both fat mass and fat-free mass. Thus, BMI is not an accurate depiction of one’s fat mass. Furthermore, several factors have been shown to affect BMI including age, race, physical training, et cetera. For example, BMI may not be an accurate measure of fat mass in various South Asian populations where the “thin yet fat” phenotype is prevalent (58), or in athletes where BMI overestimates adiposity (59).

Body mass and body composition are more accurate measures of adiposity. However, these are not easily accessible and expensive for large-scale studies. Nonetheless, various instrumentations have been used to measure lean and fat mass in humans and animals including dual energy X-ray absorptiometry (DEXA), bioelectrical impedance analysis (BIA), nuclear magnetic resonance (NMR), air displacement plethysmography, and doubly labeled isotope water, many of which are becoming more easily accessible in academic centers (47). Other methods to study adiposity include measuring waist and/or hip circumference, as markers of central obesity, and skinfold thickness, as a marker of subcutaneous or visceral adiposity (triceps, subscapular biceps, abdomen). However, the lack of accuracy and reproducibility of such measurements limits their use (47, 58). More accurate, cost-effective, and easily attainable and reproducible measures of adiposity should continue to be developed to improve the validity of these studies and should be considered an important limitation of studies of obesity in children and adults conducted thus far.

While the WHO and CDC have a clear definition of obesity from a macroscopic standpoint, on a cellular and physiologic level, obesity is an interplay of complex factors involving several organ systems. Importantly, although fat accumulation is reflective of the obese phenotype, a relative decrease in lean mass, which contributes to BMR, plays an important contribution to the development of obesity.

### The role of fat free mass in the conditioning of obesity

While measures of body weight and adiposity in childhood and adulthood are associated with one’s perinatal metabolic environment, the role of fat-free mass and BMR in the conditioning and pathogenesis of obesity remains unclear.

It was initially hypothesized that birth weight, which contributes to obesity in adulthood, would be negatively associated with fat-free mass and adult BMR. However, several studies have shown contrary results (60–62). Weyer et al. showed that while birth weight is positively associated with both fat-free mass as well as 24-hour adult EE, it is negatively associated with sleeping metabolic rate (62). While the clinical significance of this finding with regards to the pathogenesis of obesity is unclear, more thorough investigation may be needed to assess the effects of intrauterine metabolic milieu on adult fat-free mass and BMR. Further, the use of birth weight itself as a proxy measure for intrauterine nutritional status, is highly contentious. Perhaps more appropriate, future studies will determine the association between postnatal factors such as breastfeeding and early infancy weight gain, and adult fat-free mass to gain a better understanding of the role of BMR in the pathogenesis of obesity.

Thus far we have (1) discussed the global impact of childhood obesity, (2) defined obesity and factors which contribute to its pathogenesis including central and peripheral regulation of appetite, adiposity and BMR, (3) introduced the concept of metabolic conditioning and its role in the pathogenesis of obesity, and (4) presented data that supports the phenomenon of programming, which show an association between *in utero*, postnatal and early infancy metabolic factors and long-term obesity risk. In the next section, we will discuss *how* early developmental metabolic conditioning contributes to adult obesity risk.

## Mechanisms of metabolic conditioning of obesity and cardiovascular disease risk

### The conditioning of obesity in response to maternal undernutrition vs maternal obesity

The concept of metabolic programming for obesity was first demonstrated preclinically in the 1960s, although the term “metabolic programming” would be coined a few decades later (46). Robert A. McCance, an English scientist, found that overfeeding rats during the early postnatal period increased their body size in adult life. A similar outcome was demonstrated in male infant baboons by Lewis et al, where feeding a nutrient-enriched formula, with 30% excess calories, resulted in increased mesenteric and omental fat depots at 5 years (46, 63).

The Dutch famine study linked the metabolic environment of the fetus and later obesity risk with the specific gestational period of maternal undernutrition. While human fetal adipose depots develop between 14-24 weeks of gestation, they undergo significant expansion only in the third trimester, which is dependent on fat cell replication (64, 65). On the other hand, hypothalamic neurons develop by 15-18 weeks’ gestation and hormone activity in the hypothalamic-pituitary-adrenal axis can be seen as early as 8-12 weeks’ gestation and continues to mature in subsequent weeks (66). In the cohort exposed to poor nutrition in the third trimester, it is possible that the lack of maternal caloric intake restricted expansion of fetal adipose tissue, resulting in the programming of adipocytes with decreased propensity for replication, even in the presence of sufficient caloric intake. On the other hand, children exposed to nutrient deprivation in the first two trimesters, were programmed for increased responsiveness to caloric cues, cravings, or appetite due to insufficient energy substrate availability during the critical development period of the hypothalamus.

Malnutrition during the first two trimesters results in a “thrifty” phenotype, commonly known as the “predictive adaptive response”. Such conditioning becomes maladaptive in the presence of sufficient or excess nutrition later in childhood, adolescence, or adulthood. It is this mismatch between developmental conditioning and paradoxical exposures later that ultimately lead to adverse health outcomes and the risk for non-communicable disease. When the environment changes, the conditioning becomes nonadaptive. We hypothesize that a more thorough understanding of the temporo-spatial progression of obesity from early development will aide in the discovery of therapeutic interventions to prevent or reverse these maladaptive processes.

The concept of mismatch has been studied more comprehensively in preclinical models (21, 67–69). Using a rat model, Vickers et al. showed that maternal undernutrition during pregnancy promotes obesity and metabolic syndrome in male and female offspring, that is worsened by high-fat diet (HFD) feeding in the postnatal period (68). Administering leptin in the early neonatal period of 3-13 days, a critical period characterized by a high degree of developmental plasticity, reversed this phenotype (68). Gluckman et al. showed that leptin treatment in this critical postnatal period modified the transcription and methylation of genes expressed in the liver that regulate whole-body energy homeostasis including peroxisomal proliferator-activated receptor alpha (*PPARα*); however, the metabolic “program” and direction of these changes depended on the initial perinatal environment (21). Using the same rat model of maternal undernutrition with postnatal hypercaloric nutrition, Vickers et al. also showed that IGF-1 treatment alleviated hyperphagia, obesity, hyperinsulinemia and hyperleptinemia in offspring from malnourished dams (69).

This concept of developmental mismatch, however, does not apply when the exposure remains consistent. While maternal *undernutrition* may condition a “thrifty phenotype” resulting in offspring who develop obesity with exposure to high caloric diet later in life, how does maternal *obesity* result in obese offspring? One could hypothesize that like the evolutionary anticipation of possible future undernutrition in offspring born to malnourished mothers, offspring born to obese mothers should also develop a “predictive adaptive response” for future *overnutrition* and thus have *reduced* propensity for adipocyte replication and *increased* levels of satiety with feeding. However, several epidemiological studies, discussed previously, have shown the contrary: maternal obesity *increases* adiposity in offspring and programs later obesity possibly through obesogenic modifications in appetite regulation and energy metabolism (35–37, 42, 43). Preclinical studies have demonstrated the effects of maternal obesity on offspring adiposity phenotype (70–73). For example, Chen et al. noted that HFD feeding in female rats resulted in increased body weight and adiposity, hyperlipidemia and glucose intolerance in male pups at postnatal day 20 (71). White et al. showed that rat offspring at 18 weeks of age from dams fed a HFD prior to pregnancy, weighed significantly more than offspring from dams fed a normal diet (74).

**Figure 2:**
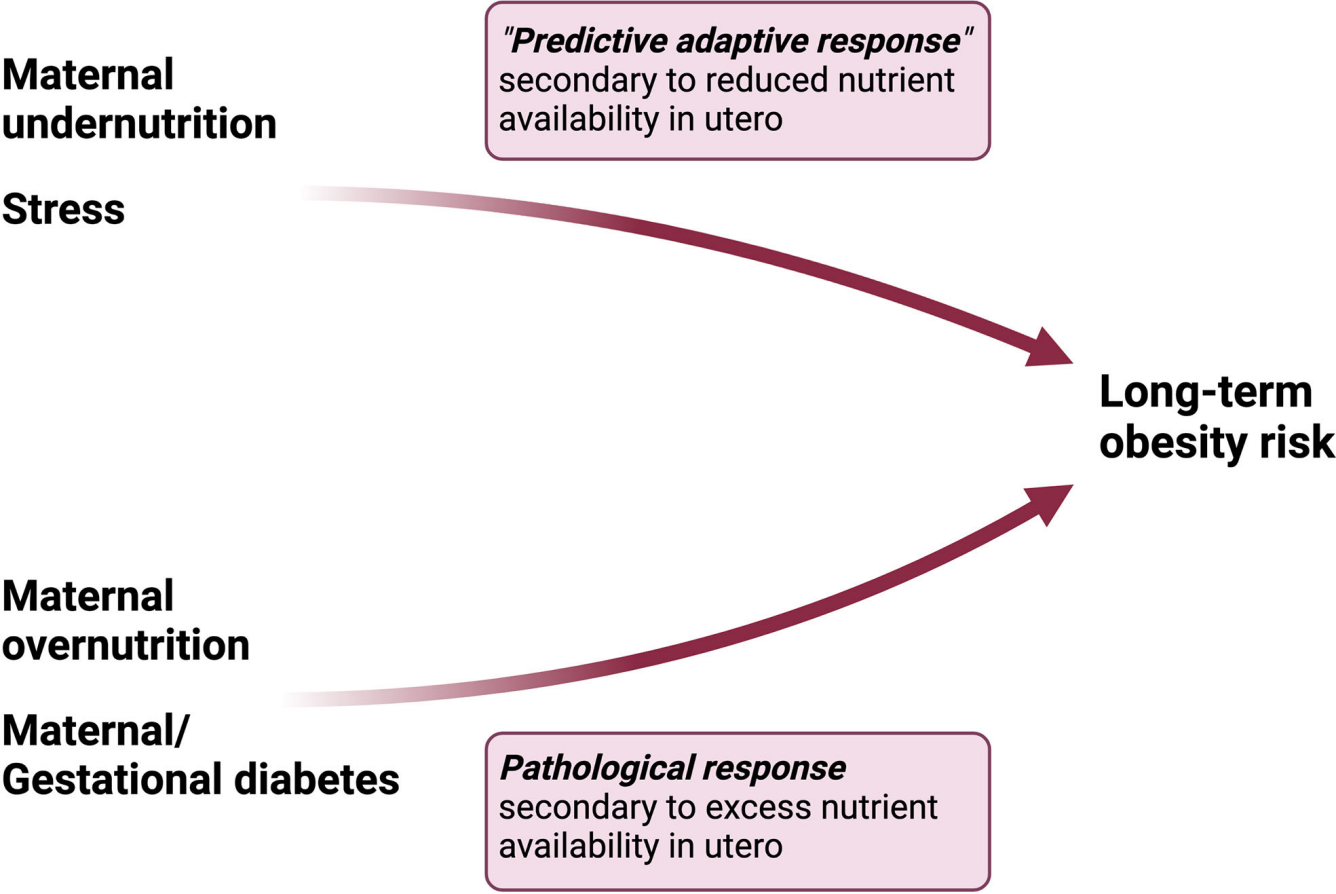
Differential pathways to obesity in response to maternal undernutrition vs overnutrition. Maternal undernutrition is hypothesized to condition obesity through a “predictive adaptive response,” in which the fetus programs its homeostatic set points in anticipation of persistent caloric deficit. However, the adaptive response becomes maladaptive in the setting of a mismatch between the anticipated environment and paradoxical exposures later in life. On the other hand, maternal overnutrition activates a pathological response in the fetus characterized by dysregulation in hormonal pathways involved in energy homeostasis.

Hanson and Gluckman in their comprehensive review of the DOHaD hypothesis, argue that maternal undernutrition (an integral thread throughout human history) has been recognized, evolutionarily, as harmful for normal development thus requiring a predictive adaptive response in order to produce fertile, viable offspring (17). Maternal obesity, on the other hand, is a relatively newer “stress” which is not yet recognized in our collective epigenetic or genetic code as maladaptive to development, since it was vanishingly rare only 10 generations ago, and the majority of offspring from mothers with obesity are viable into adulthood and capable of reproduction (17). In contrast to maternal undernutrition, maternal obesity likely plays a *pathophysiological* role in the development of obesity in offspring by altering the metabolic milieu of intrauterine life (**Figure 2**). We submit that altered maternal levels of metabolic substrates and hormones in the setting of obesity, which contribute to maternal insulin resistance, oxidative stress and inflammation, cause perturbations in glucose and lipid homeostasis and feeding circuitry in the perinatal period, ultimately contributing to long-term obesity risk. These pathophysiological factors will be discussed in detail in the next section.

### Maternal obesogenic factors that mediate the conditioning of obesity

#### Circulating metabolic substrates

Maternal obesity is associated with elevated levels of metabolic substrates including free fatty acids (FFAs), glucose and altered lipoprotein profile. FFAs and glucose can independently contribute to fetal overgrowth due to increased availability of substrates (75). Development of maternal insulin resistance and a proinflammatory state in the setting of hyperglycemia and hyperlipidemia may also contribute to an abnormal metabolic transition in early postnatal life (75). Further, exposure to elevated metabolic substrates may cause direct glucotoxic and lipotoxic effects on placental proteins, which in turn have been shown to contribute to oxidative stress, endoplasmic reticulum (ER) stress and proinflammatory pathways affecting overall placental function (76). In response to increased placental transport of glucose to the fetus, fetal hyperinsulinemia develops which is hypothesized to contribute to obesity conditioning in the offspring (77). Intrahypothalamic administration of insulin to rat pups during early development of central feeding circuitry increases body weight, impairs glucose tolerance, and promotes development of obesity and diabetes later in life (78), emphasizing its role in body weight regulation in addition to being a potent growth factor (79).

#### Circulating hormones

##### Insulin and leptin

Maternal obesity is associated with hyperinsulinemia, however maternal insulin does not cross the placenta (75, 80). While the pregnant state is naturally associated with peripheral insulin resistance to maximize glucose availability for the developing fetus, pregnant women with obesity have 50-60% higher postprandial insulin levels and are more glucose intolerant than those without obesity. Regardless, the placenta remains normosensitive to insulin in women with obesity, thus, increased placental insulin activation, along with leptin, has been shown to promote mTOR signaling leading to increased glucose and amino acid transport across the placental barrier and fetal overgrowth (81).

It is unclear which components of abnormal glucose homeostasis associated with maternal obesity – maternal hyperglycemia, hyperinsulinemia, or insulin resistance – contribute to the dysregulation of metabolic homeostasis in offspring and future obesity risk. As discussed before, the HAPO study showed that maternal hyperglycemia, in the absence of overt diabetes, increases frequency of LGA and fetal hyperinsulinemia (38), aligning with the concept that increased glucose availability and placental uptake contributes to fetal overgrowth (75). Although insulin does not cross the placental barrier, maternal hyperinsulinemia may exert effects on the placenta allowing for increased fetal nutrient uptake. While difficult to isolate the effects of maternal insulin resistance without overt diabetes on outcomes in humans, using a heterozygous knockout mouse model for insulin receptor, Carmody et al. studied the effects of maternal insulin resistance with or without maternal HFD on progeny. Maternal insulin resistance alone did not affect body weight, body composition, glucose homeostasis or expression of hypothalamic neuropeptides that regulate feeding behavior, suggesting that the intersection of maternal insulin resistance with HFD feeding is required for the metabolic programming of obesity (80). Future clinical and preclinical studies are required to isolate the individual contributions of maternal hyperglycemia, hyperinsulinemia, and insulin resistance on perturbations in energy homeostasis and offspring obesity risk.

Leptin is produced by adipose tissue, and circulating levels in weight stable adults correspond to the degree of adiposity (82). At the placental level, leptin has been shown to increase system A amino acid transport activity and stimulates the release of proinflammatory cytokine IL-6, possibly contributing to abnormal placental function (75). In the hypothalamus, insulin and leptin modify expression of neuropeptides that regulate appetite and feeding behavior. Specifically, they increase expression of anorexigenic (appetite-suppressing) precursor of alpha melanocyte stimulating hormone (α−MSH) pro-opio melanocortin (POMC) and decrease expression of orexigenic (appetite-promoting) neuropeptide Y (NPY), overall functioning to suppress appetite (82). Thus, with worsening adiposity and hyperglycemia, increasing circulating levels of leptin and insulin aid to maintain the homeostatic set point by suppressing appetite. Ensuing central and peripheral resistance to these anorexigenic hormones both characterizes and contributes to obesity and metabolic syndrome. Although not extensively studied in models of metabolic programming, insulin and leptin resistance in offspring exposed to maternal HFD *in utero* may also contribute to deregulation in energy homeostasis (73, 83–85). In a rat model, Gupta et al. showed that maternal HFD feeding *in utero* increased expression of leptin long receptor (*ObRb*) and insulin receptor b-subunit (*Ir-β*) in the offspring. The downstream leptin and insulin signaling components including signal transducers and activators of transcription-3 (STAT3) and insulin receptor substrate-2 (*Irs2*), respectively, were decreased (73). Morris et al. found that maternal overnutrition during gestation in Sprague-Dawley rats significantly decreased expression of ObRb, without significant alteration in STAT3 expression (85). Isolated elevated developmental leptin exposure can alter adult weight homeostasis in mice. Using an inducible leptin mouse model, Skowronski et al. expressed leptin untethered to obesity and demonstrated that leptin overexpression during the weaning period (corresponding to the third trimester in human brain development) caused the mice to be overly suspectable to obesity when subsequently presented a highly palatable diet (86).

Preclinical studies have shown that HFD feeding in rats and mice promotes maternal obesity as well as maternal hyperinsulinemia and hyperleptinemia. Offspring of HFD-fed rats have elevated levels of insulin and leptin that alters expression of neuropeptide hormones Npy and Pomc and may exert long-term impact on the structure and function of hypothalamic feeding circuits (71–74, 85). Chen et al, for example, found that offspring of HFD-fed have increased mRNA expression of appetite-promoting *Npy* but lower expression of appetite-suppressing *Pomc* at 20 days of life, which may represent an adaptive response to obesity (71).

The contrasting role of leptin in maternal models of undernutrition vs HFD feeding may be perplexing at first glance. We previously discussed that leptin administration in pups exposed to undernutrition *in utero* reversed the obese phenotype. However, in offspring exposed to a HFD *in utero*, both leptin and insulin are initially elevated in early life. The leptin surge in the perinatal period is critical for the normal development of hypothalamic circuitry regulating energy homeostasis, appetite and feeding behaviors (87). Both the *timing* and *magnitude* of that surge appears to be critical and if either absent (as in states of maternal undernutrition) or inappropriately elevated over a prolonged period of time (as in states of maternal overnutrition), an obese, hyperphagic phenotype ensues (77, 88).

These studies suggest that insulin and leptin play critical roles in maintaining whole body energy homeostasis and perturbations in their expression during critical time periods of development may impair long-term regulation of appetite and feeding behaviors, ultimately contributing to an obese phenotype later in life.

##### Insulin-like growth factor

IGF-1 and IGF-2, which are predominantly synthesized in the liver, regulate fetal growth and development, as well as carbohydrate metabolism (75). Both IGF-1 and IGF-2 deficiency in mice demonstrate prenatal and postnatal growth failure (89, 90). In a human choriocarcinoma cell line, IGF-1 was shown to promote trophoblast proliferation and stimulate glucose and amino acid transport, and IGF-2 deletion reduced mouse placental growth and passive nutrient delivery, suggesting key roles for IGFs in promoting fetal overgrowth (75, 90, 91).

##### Adiponectin

Adiponectin inhibits insulin signaling in trophoblasts. Maternal obesity is associated with *low* levels of adiponectin, which is thought to promote placental insulin signaling, fetal nutrient transport and in turn fetal overgrowth (75). Adiponectin supplementation in pregnant mice normalizes maternal insulin sensitivity, placental insulin/mTORC1 signaling, nutrient transport and fetal growth, despite minimal changes in maternal visceral adiposity (92). A long-term study in the same mouse model showed *in utero* adiponectin treatment ameliorated the obesogenic phenotype in 14-week-old adult male offspring, with significant reductions in body weight, fat mass and normalization of insulin sensitivity and hepatic steatosis (93).

#### Placental signaling contributes to the effects of maternal obesity on offspring

Maternal circulating levels of metabolic substrates and hormones, as discussed above, significantly impact placental function, likely mediated by mTOR signaling on the placenta by activating amino acid and glucose transport as well as mitochondrial biogenesis and protein synthesis, ultimately contributing to excess fetal nutrient delivery with increased risk for fetal overgrowth in obese women (75, 76).

#### Maternal obesogenic factors could be conveyed into long-term obesity risk in offspring *via* epigenetic modifications

**Figure 3:**
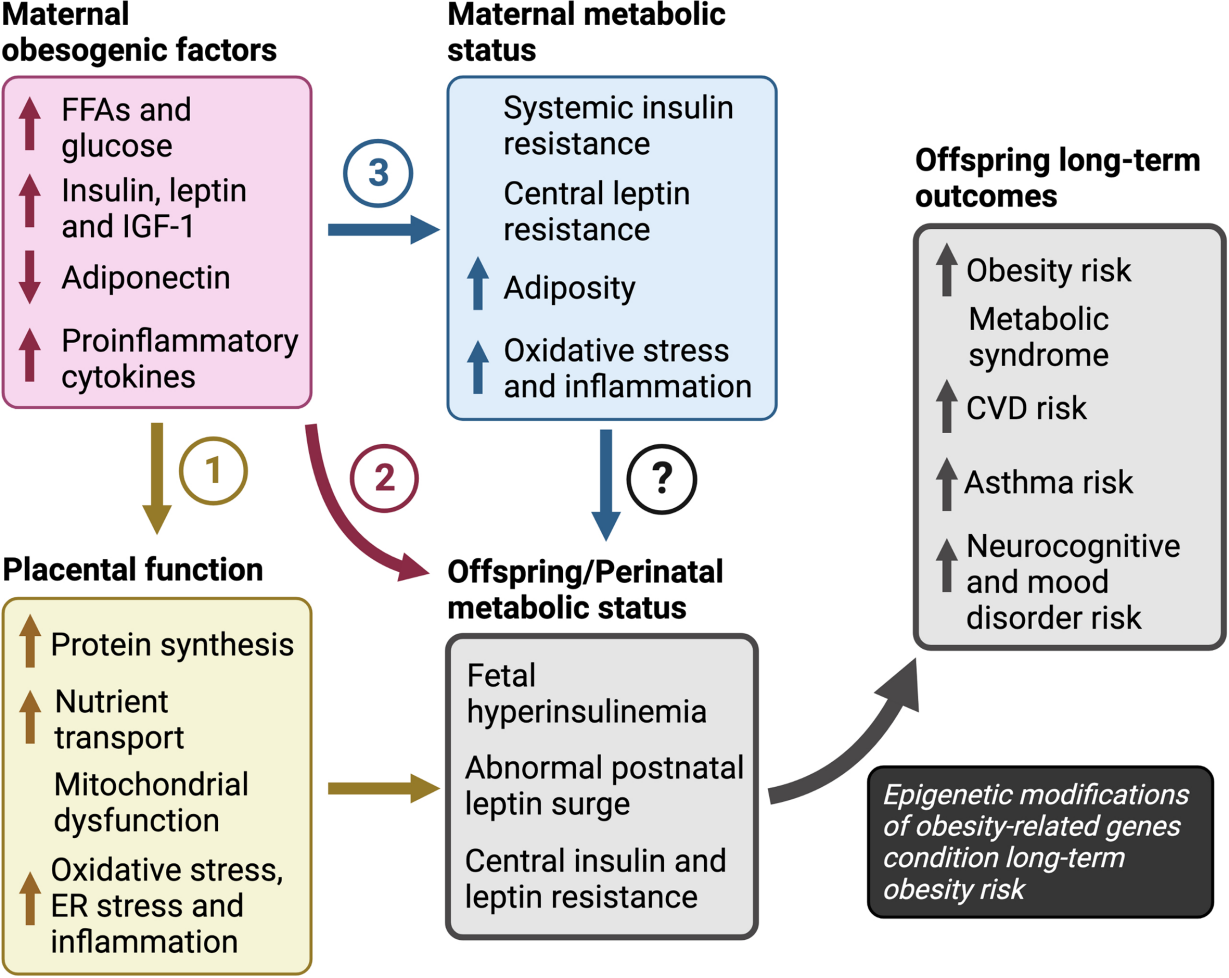
Pathways to obesity in children in response to maternal overnutrition The pathways of maternal obesogenic factors towards long-term obesity risk include: (1) dysregulation of placental function in which excess nutrients promote placental protein synthesis, mitochondrial dysfunction and increased nutrient transport, (2) increased nutrient availability, that may have a direct effect on fetal growth and development independent of changes in placental function, and (3) altered maternal sensitivity to hormones leptin and insulin. However, the roles of maternal insulin and leptin resistance, independent of maternal hyper-insulinemia or -leptinemia, are unclear and further investigation is required to determine their impact on the perinatal metabolic transition to life and outcomes in offspring. Finally, we propose that increased risk for obesity, metabolic syndrome, and atherosclerotic cardiovascular disease, depends on an abnormal metabolic status during the perinatal period in response to maternal factors. CVD; cardiovascular disease, ER; endoplasmic reticulum, FFAs; free fatty acids, IGF-1; insulin-like growth factor-1.

Several maternal obesogenic factors contribute to obesity in offspring, including glucose, FFAs, insulin, leptin, IGF-1 and adiponectin. Together, they promote maternal insulin resistance, hyperglycemia, hyperleptinemia, oxidative stress and inflammation *in utero* which perturb the development of central feeding circuitry and energy homeostasis during the perinatal transition. The ensuing fetal hyperleptinemia, insulinemia and transient neonatal hypoglycemia increase the risk for fetal overgrowth and LGA (**Figure 3**). But how does this short-term dysregulation in energy homeostasis during the perinatal period affect long-term risk for obesity? We posit that the *in utero* environment caused by maternal obesity, and the concomitant short-term perinatal aberrations in energy homeostasis, translate to long-term obesity risk through epigenetic modifications in both central and peripheral markers that regulate energy homeostatic set points.

Epigenetics is the study of heritable changes in gene expression secondary to chromatin modifications such as DNA methylation, as well as histone and miRNA modifications, without an actual change in DNA sequence (94). DNA methylation contributes to gene expression and is considered one of the primary mechanisms of cellular memory. Specifically, methylation of the 5’ position of cytosine by DNA methyltransferases to form 5-methylcytosine (5mC) impedes binding of transcription factors, which ultimately represses transcription of the gene. On the other hand, hypermethylation of CpG islands can promote transcriptional activation (95). Such epigenetic changes to gene expression are critical for normal cell differentiation and development and maturation of organ systems early in life (96, 97). In response to an abnormal metabolic environment *in utero* and in the perinatal period, alterations in epigenetic modifications of obesity-related genes can have lasting effects on gene expression, ultimately contributing to obesity risk (21, 98–100).

Many epigenetic modifications have been identified to play a role in the pathogenesis of obesity. For example, altered methylation patterns have been seen in genes that regulate systemic energy homeostasis such as *HIF3A* (hypoxia-inducible factor 3A), *LEP* (leptin) and *ADP* (adiponectin), as well as *POMC*, *PPARα*, *PGC1α*, *IGF-2*, *IRS-1*, *IL-6* (other genes involved in whole-body lipid and carbohydrate metabolism, insulin signaling and inflammation). Hypermethylation patterns have also been identified in genes related to the circadian rhythm *–CLOCK*, *BMAL1* (in leukocytes and adipocytes). Histone modifications of genes related to adipogenesis and adipocyte differentiation include *PREF-1*, *C/EBPα/β*, *PPARγ* and *aP2*. Finally, expression of certain miRNAs are associated with increased fat storage in adipocytes and may upregulate adipogenesis in obese states. For example, miR-26b promotes proliferation of preadipocytes and expression of *PPARγ* (94, 95). Epigenetic changes can be stable in an organism from one cell to its progeny through cellular division and mitosis, but also across generations and, are thus, considered inheritable. For example, the DNA methylation status of the Agouti focus, responsible in determining fur coloration, is passed on transgenerationally through maternal inheritance (94, 95).

An elegant study performed by Masuyama et al. showed that exposure to a HFD *in utero* results in an obese phenotype in offspring over three generations through the maternal line along with elevated plasma and tissue level expression of leptin and reduction in adiponectin (101). The adiponectin level was driven by increased acetylation and decreased methylation of H3K9 at the promoter region of adiponectin gene in offspring of HFD-fed mice. In contrast, monomethyl H4K20 levels were increased in the *Lep* promoter region of these same offspring. Reversal of the phenotype and epigenome was only possible by chow fed diet feeding in three subsequent generations (101).

#### Obesity conditioning occurs primarily during critical developmental periods

The critical periods of metabolic programming extend from fetal, perinatal, as well as early postnatal stages (17, 102). During the fetal stage and metabolic transition to postnatal life, there is a high degree of plasticity and epigenetic modifications have been demonstrated in key regulatory genes which affect the development of the hypothalamus and its peripheral connections (66, 94, 95). Further in mammals, significant structural and functional development of adipose tissue, liver and gut occurs after birth. Thus, the postnatal period serves as an important continuation of the fetal phase (102).

For example, in rats and mice, the hypothalamic-pituitary axis is immature at the time of birth and undergoes maturation in the first 2 weeks of life (66, 87, 94, 103). Variations in DNA methylation between hypothalamic and non-neuronal cells are established in the postnatal period (104). In humans, DNA methylation of prefrontal cortex involved in appetite control, satiation, and food craving, increases steadily overtime, exhibiting prolonged postnatal maturation (94, 102–104).

In rodents, white adipose tissue (WAT) is minimal at the time of birth and undergoes postnatal maturation (102). Epididymal WAT is composed of progenitor cells that lack the capacity to differentiate from postnatal days 1 to 4, following which dividing cells are observed. In humans, although WAT is present from the second trimester of gestation, significant growth occurs from birth to 6 weeks of age, during which time body fat doubles from approximately 10% to 20%. After birth, both adipocyte size and number increases accounting for the large adipose tissue expansion (64, 65, 94, 102).

Finally, the liver acquires its metabolic functions and undergoes most of its methylation postnatally (102). Indeed, regulatory regions, including promoters and enhancers of genes involved in lipid and glucose metabolism in the liver, undergo programmed active DNA demethylation in a time-dependent manner after birth. This process has been shown to be partly dependent on the activity of ten-eleven translocation dioxygenases, otherwise known as “TET” enzymes (*Tet2* and *Tet3*), which promote DNA demethylation (105).

Despite the high degree of plasticity during the perinatal period, several other factors may result in epigenetic modifications that alter the long-term ability to mobilize fat beyond these critical developmental periods. These include environmental toxins known as “obesogens,” changes in gut microbiota and excess dietary intake during early childhood (94, 102). We argue that epigenetic changes are not exclusive to the perinatal period and that these changes may occur along any time in response to various behavioral and environmental factors.

Epigenetic modifications, in response to early environmental exposures, are known to persist into adulthood. For example, the cohort of children from the Dutch Hunger Winter exposed to maternal caloric restriction in the first trimester of gestation had less DNA methylation of the IGF2 gene compared with their unexposed siblings six decades later (94, 106). However, epigenetic changes are not confined by longevity or permanence. Indeed, contrary to the initial understanding of epigenetic mechanisms in the programming of obesity, we now know that these changes are highly dynamic and reversible.

If one’s metabolic milieu is altered in favor of weight loss, the epigenetic alterations in DNA methylation may be reversible (94). As discussed previously, the methylation patterns of the promoter regions of leptin and adiponectin genes in mice exposed to a HFD *in utero*, were reversed in three generations upon transitioning to a normal chow diet (101). Further, dietary changes, exercise, and surgical interventions may reverse epigenetic changes. For example, maternal bariatric surgery and associated weight loss improved the metabolic profile of children during their adolescent period compared to their siblings born prior to weight loss surgery, with decreased birth weight, obesity incidence, blood pressure and adiposity, as well as improved insulin sensitivity and lipid profile (107, 108). Siblings born after weight loss surgery exhibited differences in epigenetic modifications in genes related to glucose homeostasis, insulin resistance, inflammation, and vascular disease (99).

The discovery of ten-eleven translocation dioxygenases enzymes, which reverse DNA methylation, has made the mechanistic underpinnings of methylation reversal a possibility (102, 105). “Ten-Eleven Translocation dioxygenases” or TETs convert 5-methylcytosine (5mC) to 5-hydroxymethylcytosine (5hmC), that can then undergo serial oxidation resulting in active DNA demethylation *via* removal and substitution with unmethylated cytosine *via* base excision repair. Indeed, 5hmC is detected in several different cell types near regulatory regions (promoters/enhancers) and positively correlates with gene expression (105).

### Epigenetic modifications during different time periods of development affect later obesity risk

#### Prenatal period

The currently known epigenetic pathways that contribute to the conditioning of obesity include variations in methylation status of obesity-related genes associated with differences in adiposity and body composition in infancy and beyond. An elegant longitudinal clinical study measured the methylation status of CpGs in the promoters of candidate genes from umbilical cord tissue of neonates obtained at birth along with clinical measures of adiposity at 9 years of age (98). Higher methylation level of retinoid X receptor-alpha (*RXRα*) at birth was strongly correlated with adiposity in childhood. RXRα is a transcription factor that regulates expression of genes including *PPARα* involved in whole-body lipid and glucose metabolism, insulin sensitivity and adipogenesis, suggesting an association between early life epigenetic modifications and later obesity risk.

Maternal weight loss surgery is known to reduce the risk of offspring obesity without large maternal weight loss (100, 108). Children born to mothers who underwent biliopancreatic diversion prior to pregnancy had significantly reduced odds of developing obesity from 2-18 years of age, even if the mothers remained overweight after surgery. Altered methylation patterns and expression of genes involved in glucose metabolism, insulin sensitivity, immune and inflammatory-related functions was noted in the cord blood after the surgery (99). We hypothesize that overall improved nutritional status during gestation with concomitant decreased maternal insulin resistance, hyperglycemia, hyperlipidemia, and hyperinsulinemia contribute to a healthy *in utero* environment translating to better offspring outcomes.

Preclinical studies have also shown that variations in the methylation status of obesity-related genes, in response to abnormal maternal diet *in utero*, are associated with weight gain and obesity in the offspring (109–112). Jousse et al. found that maternal undernutrition in mice during gestation and lactation produces hyperphagic offspring with higher food intake throughout life (112). Mice exposed to maternal undernutrition showed persistent changes in serum levels, mRNA expression and methylation patterns of leptin. Fetal malnutrition results in lower leptin levels with corresponding decreased adipose-specific mRNA expression, as well as long-term changes in methylation of the leptin promoter (112).

Lillycrop et al. found that maternal protein restriction in female rats during pregnancy decreased methylation of hepatic glucocorticoid receptor (*Gr*) and *Pparα*, and increased expression of mRNA transcripts in pups at 6 days of life (109). These epigenetic changes persisted after weaning (while pups were no longer on decreased protein intake). A follow-up study showed persistence of changes in methylation of *Pparα* at 34 and 80 days, corresponding to the adolescent and adult periods respectively (113). Further, folic acid supplementation either during gestation or in adolescence prevented or reversed these epigenetic modifications respectively (109, 114). As DNA methylation requires S-adenosylmethionine as a methyl donor, which is dependent upon serine, glycine, folate, and vitamin B12, epigenetic modifications may be altered by availability of these amino acids and micronutrients *in utero*. These data suggest that maternal nutritional status alters the fetal epigenome and the expression of energy homeostasis genes throughout lifespan (109, 110, 113).

Gluckman et al. showed that maternal protein restriction and post-weaning hypercaloric nutrition followed by leptin treatment from days 3 to 10 of life, reversed the obese phenotype in offspring described previously, and resulted in stable and long-term changes in the methylation patterns and expression of various markers later in life (day 170) (21). Methylation of the *Pparα* promoter was either (1) decreased by leptin treatment in offspring exposed to maternal undernutrition *in utero*, corresponding to enhanced transcript expression, or (2) increased by leptin treatment in offspring without exposure, corresponding to decreased transcript expression. Offspring transitioned to a HFD post-weaning obscured the effects of leptin on *Pparα* expression. Methylation of the *Gr* promoter, on the other hand, was elevated by leptin in maternally well-nourished offspring but unaffected in offspring exposed to maternal protein restriction *in utero*. Finally, leptin also had bidirectional effects on the expression of hepatic 11β-hydroxysteroid dehydrogenase type 2 (*11β-Hsd2*), which is an enzyme that inactivates glucocorticoids. While neonatal leptin treatment decreased 11β-Hsd2 in offspring from undernourished mothers, it increased 11β-Hsd2 in offspring from well-nourished mothers (21). Using a rat model of overfeeding in which mothers developed obesity preconception, Borengasser et al. showed altered methylation patterns and corresponding expression of adipogenic genes in white adipose tissue of offspring (115). Offspring of obese dams showed decreased methylation of *C/ebp-β*, *Zfp324* and *Pparγ*, which are involved in the development of adipose tissue, as well as corresponding increased expression of their downstream targets, ultimately resulting in enhanced adipogenic differentiation and greater adiposity in offspring. Fernandez-Twinn et al. showed that HFD-feeding during gestation in a mouse model produced offspring with unchanged body composition, energy expenditure, activity levels or glucose tolerance at 8 weeks of age; however, fasting insulin levels were significantly higher compared to mice from chow-fed mothers (116). Interestingly, protein expression of Irβ was downregulated in epididymal fat in offspring from HFD-fed mothers, in addition to further downstream markers of insulin signaling including Irs-1, Pi3k, Akt1 and Akt2. Further miR-126 was found to regulate the expression of Irs-1, with a potential role in the epigenetic programming of insulin resistance.

In a genetic mouse model of maternal obesity and diabetes, offspring at 12 weeks of age displayed elevated liver lipid contents, as well as elevated serum leptin (117). Offspring exposed to maternal obesity and diabetes and subsequently challenged with a western-style diet gained significantly more weight and demonstrated glucose intolerance and insulin resistance, compared to offspring from control mice. These phenotypic changes corresponded to variations in hepatic gene expression, specifically those regulating mitochondrial activity such as *Atpase6* and *Cytb*. The epigenetic alterations were not restricted to genes involved in energy homeostasis but were widespread relating to embryonic and tissue/organ development.

In another mouse model of diet-induced maternal obesity, significant alterations in histone acetylation and methylation of hepatic transcripts involved in lipid metabolism were observed in offspring exposed to maternal overnutrition during gestation, which persisted into adulthood (5 weeks of age) (118). Specifically, *Sirt1*, which encodes a histone deacetylase with functions in lipid metabolism and obesity (serving as a marker for cellular energy levels), was significantly reduced in offspring exposed to maternal obesity and persisted into adulthood. Further, differential histone modifications were also seen in *Pparα*, *Pparγ*, *Rora* and *Rxrα* in the offspring from obese mothers that were not sustained at 5 weeks.

#### Postnatal period

Several human studies show a differential methylation pattern of *LEP* promoter in breast fed infants (102, 119–122). A cross-sectional study in 120 Dutch children found that longer breastfeeding duration reduced the average CpG methylation of *LEP* promoter in peripheral blood and was negatively associated with plasma leptin and infant BMI at an average age of 1.4 years (120). In a prospective cohort study, Pauwels et al. noted that every extra month of breastfeeding was associated with a 0.217% increase in *RXRα* CpG2 methylation (95% CI: 0.103, 0.330; p < 0.001). Similarly, increased levels of CpG3 methylation of *LEP* promoter was seen with 7-9 months of breastfeeding (6.1%) compared to 1-3 months (4.3%; p = 0.007) that did not persist at 10-12 months. However, infant weight and BMI-for-age at 1 year was significantly lower in children who were breastfed for 10-12 months, suggesting that breastfeeding-mediated epigenetic modifications in *RXRα* and *LEP* may have a role in childhood obesity. In a longer prospective cohort study, Sherwood et al. demonstrated that *LEP* methylation at four CpG sites at 10 years of age was associated with exclusive breastfeeding (121).

Rodent models also show an association between postnatal feeding and differential methylation patterns of obesity-related genes. Rats overnourished during the breastfeeding period by reduction in litter size have increased growth rate, hyperinsulinemia and hyperleptinemia, and overweight throughout life with central leptin and insulin resistance in adulthood (123–125). In another rat model of postnatal overnutrition, Mahmood et al. demonstrated hyperinsulinemia persistent in the post-weaning period in newborn rats fed a high-carbohydrate milk formula (126–128). These animals developed hyperphagic obesity in adult life on a standard chow diet along with associated epigenetic alterations in *Npy* and *Pomc* (128). While *Npy* mRNA expression was increased in the hypothalami of both 16- and 100-day old high-carb fed rats, with increased methylation of specific CpG positions and histone acetylation, *Pomc* expression was reduced, accompanied by a decrease in histone acetylation.

### Postnatal exposures can reverse the gestational programming of obesity

Several studies suggest that postnatal exposures may have a greater impact compared to *in utero* exposures (100, 129). Using a rat model of diet-induced obesity in which Sprague-Dawley rats were selectively bred for diet-induced obesity and resistance, Gorski et al, found that obesity-prone pups fostered to lean dams at birth remained obese in spite of reduction in food intake and gradual improvement in insulin sensitivity compared to obesity-prone pups fostered to obese dams (129). They noted an upregulation in hypothalamic expression of leptin receptor (*Lepr-b*) and insulin receptor (*Ir*), suggesting improved leptin sensitivity. On the other hand, lean pups fostered to obese dams during lactation develop obesity and insulin resistance when fed high-caloric diet, with a decrease in expression of *Lepr-b* and *Insr*.

Obesity programming may also have a role in the development of the neuronal projections relevant to energy homeostasis. In a study of offspring born to dams fed a HFD during lactation, Vogt et al. observed no differences in the expression of *Pomc*, *Npy* and agouti-related peptide (*AgRP*). However, they noted impaired *Pomc*- and *Agrp*- neuronal projections to target sites within the hypothalamus (130).

Finally, as discussed previously, rat models of maternal food restriction during pregnancy cause intrauterine growth restriction in offspring who later show rapid catch-up growth with adult obesity and metabolic syndrome (21, 109, 111–113). This phenotype can be prevented through continued maternal undernutrition during the lactation period, where availability of nutrients is also limited to the newborn (131, 132). Tosh et al. showed that maternal food restriction during both pregnancy and lactation decreased hepatic mRNA and protein expression of *Igf1*, that persisted at 9 months of age (132). Transitioning the dams to a regular diet during the lactation period rescued the expression of *Igf1* in the adult offspring, both in transcript expression and serum levels.

## Discussion

### Future directions to prevent the evolution of obesity from conception

#### Summary and additional remarks

Early exposure to maternal obesogenic factors from conception “programs” long-term obesity risk through epigenetic modifications in genes involved in energy homeostasis. While there are several risk factors for childhood obesity, maternal overnutrition during pregnancy has consistently been shown to alter the fetal epigenome. The effects of epigenetic alterations often persist into adulthood, contributing to the long-term obesity risk in offspring. Observational studies suggest that maternal diet, rather than maternal weight in isolation, may be the driver of the epigenetic modifications during the perinatal period. Previously described study of siblings born before and after maternal bariatric surgery found that significant weight reduction or normalization of BMI was not necessary to reduce the risk of obesity in offspring, although the systemic effects of bariatric surgery on programming of feeding behavior and whole-body metabolism in offspring remain unclear (108). Further, the Dutch famine study and studies on rodent models have shown that maternal undernutrition also predisposes to longer-term obesity in offspring (21, 82, 106, 109, 111–113). Thus, we hypothesize that the *in utero* metabolic environment, which is significantly influenced by maternal diet, determines the predisposition to obesity in offspring.

In this review, we have described several markers that have been shown to be susceptible to epigenetic modifications during critical developmental periods to promote an obesogenic phenotype during lifespan. These obesity-related genes are primarily expressed in the hypothalamus, liver, adipose tissue, and pancreas and serve as potential therapeutic targets for both the treatment and prevention of obesity, summarized in **Table 1**.

**Table 1:** Tissue-specific pathways and genes involved in the metabolic programming of obesity.

Tissue	Obesogenic pathway	Target gene(s)	References (preclinical)	References (clinical)
**Hypothalamus**	Central feeding dysregulation	*NPY*	(71, 73, 84, 124, 127)	
		*POMC*	(71, 73, 84, 127, 129)*	
		*AgRP*	(73, 129)*	
		*MC4R*	(84)	
	Central leptin resistance	ObRb,	(73, 84), 122)**	
		Stat-3		
	Central insulin resistance	*IR-β, STAT-3 IRS-2*	(73, 123)**	
**Adipose Tissue**	Adipogenesis	*C/EBP-β*	(114)	
		*PPARγ*	(114)	
	Peripheral insulin resistance	*IR-β, IRS-1, PI3K, AKT1/2*	(115)	
		*ADP*	(100)	
	Abnormal postnatal leptin surge	*LEP*	(21, 67, 68)	
	Persistent hyperleptinemia	*LEP*	(100)	(119, 120, 121)
**Pancreas**	Fetal hyperinsulinemia	*INS*	(72, 78)	
**Liver**	Lipid and glucose metabolic dysregulation	*PPARα*	(21, 108, 110, 112, 113, 117)	
		*RXRα*	(117)	(97, 121)
		*SIRT1*	(117)	
		*GR*	(21, 108, 110, 113)	
	Abnormal growth and development	*IGF1*	(69)	
		*IGF2*	(105)	

Several markers that regulate energy homeostasis have been found to contribute to the epigenetic programming of obesity. Here, we have included data primarily from animal models, which have identified changes in tissue-specific (1) epigenetic regulation and/or (2) mRNA/protein expression of these genes in response to either maternal undernutrition or overnutrition during the prenatal and/or postnatal periods. We have further stratified these markers into tissue-specific obesogenic pathways. The clinical data referenced, notably, did not investigate tissue-level changes as the human samples obtained were of blood, buccal swab or placental tissue. Nonetheless, they too were stratified into tissue-specific obesogenic pathways based on the known functional role of the marker and interpreting the results of the clinical study within the context of preclinical data referenced here investigating the same marker.

*This study did not show changes in expression or methylation patterns of POMC or AgRP but rather observed altered neuronal projections to target hypothalamic sites in response to postnatal maternal HFD feeding.

**Central leptin or insulin resistance was evaluated in these studies by measuring the firing rate of arcuate neurons in response to exogenous leptin or insulin, respectively, in rats postnatally overnourished, not by measuring the expression or methylation patterns of downstream signaling molecules.

A preclinical study on the long-term effects of epigenetic changes in offspring in response to maternal undernutrition showed that some epigenetic modifications do not persist into adulthood (121). While it is unclear why some genes are more susceptible to reversal of epigenetic modifications compared to others, we argue that the *effects* of those initial epigenetic alterations likely contribute to the cumulative risk for obesity throughout life. Epigenetic alterations sustained during early critical developmental time periods can be considered as the “first-hit” (of many) in the pathogenesis of obesity. While, in the setting of maternal overnutrition, hypothalamic circuitry has been re-programmed towards lower levels of satiety and increased appetite, peripheral metabolism has been conditioned towards increased lipid storage and insulin resistance. Thus, we argue that the homeostatic set points which are established in early life initiate the temporospatial evolution of obesity and contribute to cumulative risk of metabolic syndrome, even if some epigenetic modifications which conditioned those set points may not persist. The mechanisms behind this hypothesis are unclear, and further investigation is required to understand the factors mediating the changes caused by these epigenetic modifications throughout lifespan.

#### Transgenerational transfer of metabolic risk

We have outlined evidence from human and animal studies on the metabolic risk caused by intrauterine and maternal exposures (F0 generation) to the offspring (F1 generation). The transmission of this risk to the subsequent generations resulting in transgenerational risk of metabolic disease into F2/F3 generation and beyond is possible. Epidemiological evidence suggests that exposure to maternal obesity and T2D results in higher prevalence of obesity, T2D (133, 134) and cardiometabolic risk in adult children (135), that may potentially remain in subsequent generation(s). Animal studies, that allow isolation of the gestational and the postnatal environment have established that the transmission of the risks conferred by maternal/gestational exposures may persist for up to 4 generations through both maternal and paternal germlines (136, 137). Evidence for such transmission is available in many animal species by environmental exposures. As an example, sex-specific inheritance of impact of high fat diet induced changes was demonstrated in females in F3 generation *via* paternal transmission in mice (138) that could be abolished by normal diet for three generations (101). Many similar examples are available in the review by King et al. (136) and Mohajer et al. (137) emphasizing the need and urgency of interrupting the cycle of transgenerational transfer of metabolic risk.

#### Reversing the epigenetic modifications which “program” obesity

We previously described both clinical and preclinical studies that showed a reversal of epigenetic modifications of obesity-related genes with improvement in metabolic status *in utero* or in later life (94, 99, 101). The discovery of TET enzymes made the mechanistic reversal of DNA methylation a possibility, however, how this process is activated, and under what conditions, remains unknown.

Dietary supplementation with folic acid has been shown to modulate the epigenetic modifications by altering availability of substrates for methylation of DNA, thus potentially reversing the epigenetic alterations of obesity-related genes (109). Natural compounds from dietary sources such as quercetin, curcumin, genistein, resveratrol and lycopene, may act as epigenetic modifiers (139). These compounds have been studied extensively in cancer biology, as they simultaneously possess anti-inflammatory, antioxidant and anticancer properties and may have a potential benefit in targeting the conditioning of obesity (140, 141).

Cancer and immunology research have further provided data for the use of “epigenetic drugs” (141, 142). Certain classes of epigenetic modifiers, such as histone deacetylase inhibitors (HDACi), histone acetyltransferase inhibitors (HATi), DNA methyltransferase inhibitors (DNMTis), histone demtheylating inhibitors (HDMis) and sirtuin-activating compounds (STACs), are currently being used for the treatment of cancer, urea cycle disorders, epilepsy, and hypertension. It is possible that these or other similar drugs may have a potential for the treatment and prevention of metabolic diseases such as obesity and diabetes. Early studies of synthetic and natural compounds including resveratrol (STAC), curcumin (HATi), hydralazine (DNMTi), valproic acid (HDAi) and sodium phenylbutyrate (HDAi) in obesity and diabetes are promising (140).

#### Limitations of epigenetic studies

Evaluating a cause-and-effect relationship between early life epigenetic modifications in response to maternal exposures and later obesity risk in *humans* is challenging for two primary reasons. First, most clinical studies investigating the epigenetic programming of obesity use blood, easy-to-obtain epithelial (i.e., buccal swabs) or placental samples (i.e., cord blood or tissue) to measure epigenetic modifications during the perinatal period and later in childhood. There is, for obvious reasons, limited access to central and peripheral tissues including the hypothalamus, adipose tissue, liver, and skeletal muscle which would otherwise help to more thoroughly understand the functional roles of obesity-related genes that undergo epigenetic alterations during critical time periods of development and condition obesity. Discovery of circulating epigenetic markers that more accurately reflect tissue-specific changes will be important moving forward in this field.

The second limitation of both preclinical and clinical studies investigating the role of epigenetics in the conditioning of obesity is simply the vast number of epigenetic modifications possible for one gene, which synergistically affect its expression. In other words, while most studies discussed in this review have focused on measuring differential methylation patterns of gene promoters or CpG islands, several other DNA and histone modifications shape the expression patterns of genes including histone acetylation and non-coding RNAs (i.e. microRNAs). While it is time-consuming and challenging to measure multiple epigenetic modifications, it is also unclear whether long-term expression of genes can be extrapolated based on single or few epigenetic changes that can be currently studied.

#### Lifestyle modifications remain vital in the prevention and treatment of childhood obesity

This and other prior reviews regarding the epigenetic conditioning of obesity discuss data on the contribution of *in utero* exposures on long-term obesity risk due to the abnormal “programming” of energy homeostatic set points likely making it more challenging to initiate and maintain weight loss. We hypothesize that lifestyle modifications may lead to better outcomes when applied earlier in life during periods of developmental plasticity. Preclinical studies suggest that improved nutrition during lactation can reverse and/or prevent the consequences of poor maternal diet *in utero*, although how maternal diet and exposures during postnatal feeding affect breastmilk content requires further investigation. Nevertheless, although lifestyle modifications in adults are not successful in promoting long-term maintenance of weight loss, we hypothesize that maternal lifestyle modifications either before pregnancy, during gestation and/or in the postnatal period, will improve long-term outcomes *in children* and reduce their risk for obesity in later life.

Further, although the perinatal period likely has the highest degree of central and peripheral plasticity; central feeding circuitry, peripheral innervations and metabolically active tissues are still susceptible to epigenetic modifications, and the consequences of *in utero* exposures may likely be reversed when lifestyle modifications are applied during childhood and even the adolescent period.

Proven to be more efficacious and successful than lifestyle modifications, maternal bariatric surgery prior to pregnancy has been shown to reduce long-term obesity risk in children; however, is typically only performed in patients with significant obesity (BMI ≥ 40 or BMI ≥ 35 in patients with significant comorbidities) (108, 143). Although less efficacious than bariatric surgery, several pharmaceutical interventions have been approved by the Food and Drug Administration (FDA) for the treatment of obesity (143–148). Of note, almost all current drug therapies *are more efficacious* in weight loss when combined with lifestyle modifications. Treatments such as GLP-1 receptor agonists, with or without other peptides, significantly improve overall metabolic status by increasing insulin sensitivity and modifying absorption of carbohydrates (149). As discussed before, improvement in maternal metabolic status appears to be more important than reduction of maternal adiposity alone to reduce long-term risk for obesity in children, although, clinically, adiposity and insulin resistance are often interdependent especially in the setting of maternal overnutrition.

We hypothesize that treatments targeting adiposity and insulin resistance simultaneously, in patients with or without overt diabetes, may be efficacious in both treating obesity and reducing offspring risk for obesity and diabetes. Increasing number of drugs, including GLP-1 receptor analogues, have been approved for treatment of obesity and diabetes in youth 12 years and older. Further, targeted drug therapy for specific genetic variants involved in the leptin melanocortin pathway are approved for children 6 years and older; ongoing clinical trials may expand this armamentarium in the future (150).

#### Clinical practice recommendations to reduce long-term risk of obesity in children

Based on data presented in this review, we recommend mothers with obesity to incorporate lifestyle modifications including consuming a well-balanced diet and physical activity prior to conception, during gestation (within reasonable limits) as well as during lactation to reduce long-term obesity risk in children.

Rapid weight gain during early infancy has been shown to significantly increase the risk for obesity later in life. We propose that breastfeeding should be encouraged in all infants, especially those of mothers with obesity and formula supplementation should be limited in breastfed infants to reduce risk of overfeeding. Further, based on the study by Huh et al. (151), the introduction of solid foods should be withheld prior to 4 months of age to avoid overnutrition in the first few months of life – a critical window of development during maturation of energy homeostatic systems setting the course for future feeding behavior and basal metabolism.

**Figure 4:**
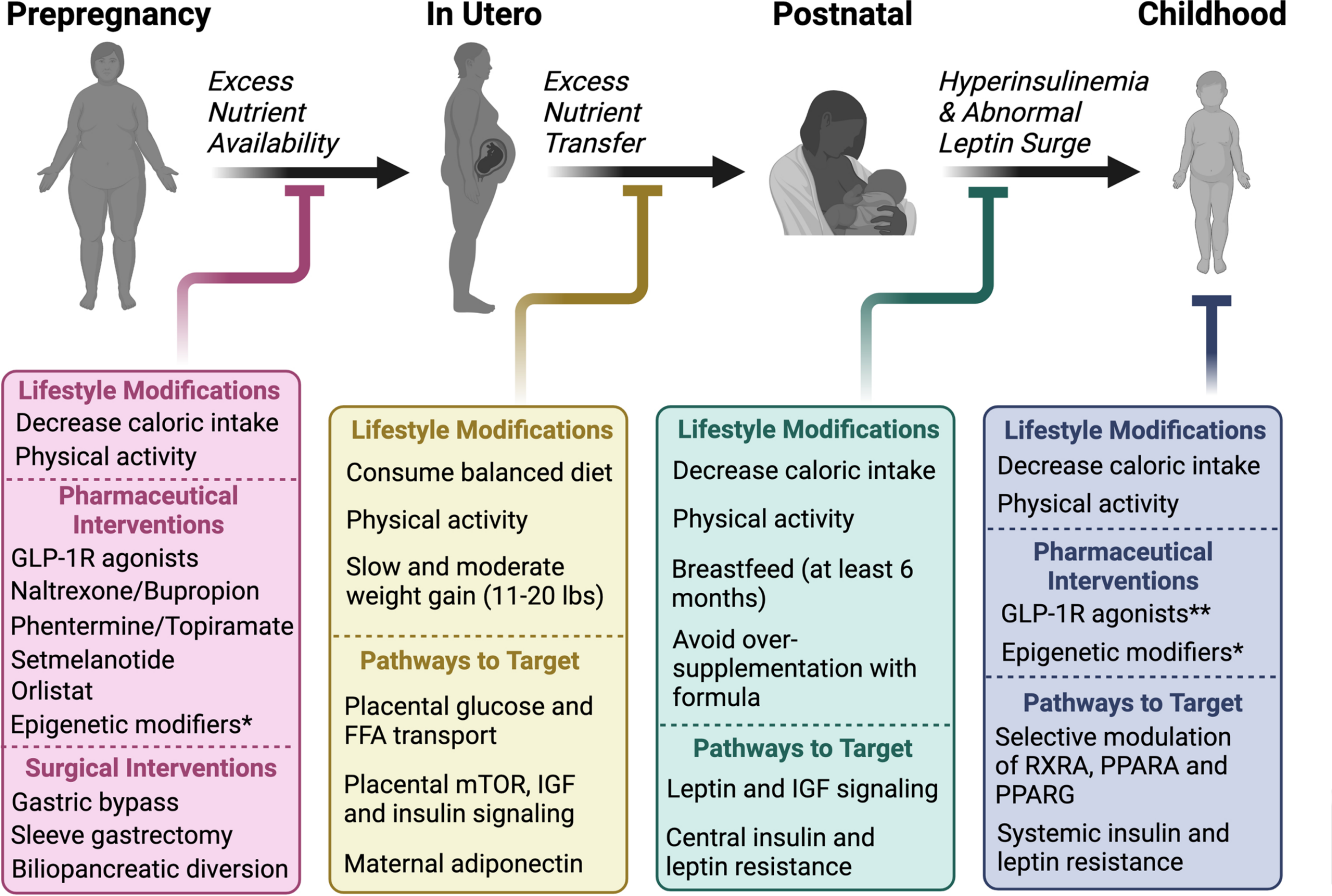
Time-dependent interventions to prevent the temporospatial evolution of obesity from prepregnancy to childhood Maternal lifestyle modifications, despite lack of effectiveness in long-term weight loss maintenance in adults, remain critical for the prevention of obesity in children. Excess nutrient availability, which concomitantly leads to an over-nourished *in utero* environment, is the initial insult in maternal obesity that predisposes children to obesity. Lifestyle modifications including a reduction in caloric intake and physical activity, as well as surgical and pharmaceutical interventions are available to ameliorate excess nutrient availability to a potential fetus. Although weight loss is not recommended while pregnant, methods to target excess nutrient transfer to the fetus include consuming a balanced diet and maintaining a moderate level of physical activity with slow weight gain (a maximum of 11-20 lbs is recommended by the NIH for pregnant women with obesity). In addition, research efforts should be focused on targeting pathways that promote nutrient transport to the fetus including energy substrate transporters, as well as mTOR, IGF and insulin signaling. Maternal adiponectin treatment in rodent models has been shown to improve metabolic status in offspring and is another possible therapeutic option in humans during this period. After birth, maternal lifestyle modifications again are critical although how these affect breastmilk content remains unclear. Nonetheless, breastfeeding for at least 6 months has been shown to reduce risk for obesity in children. Further, avoiding overfeeding and rapid weight gain during infancy is critical in reducing long-term risk for metabolic syndrome. Finally, in children with obesity, current interventions remain limited. Based on preclinical and clinical data discussed, we hypothesize that obesity treatments, which may help to reverse the epigenetic programming of obesity, may be more effective during childhood vs adulthood as obesity-related genes and signaling pathways regulating central feeding circuitry and basal metabolism are likely more susceptible to reversal of epigenetic alterations during this time. In addition to epigenetic modifiers, other pathways to target include systemic insulin and leptin, as well as RXRA, PPARA and PPARG signaling which regulate feeding behavior, as well as systemic lipid and glucose homeostasis, respectively.*Epigenetic modifiers (both natural and synthetic compounds) are not currently approved for the treatment or prevention of obesity but have excellent therapeutic potential based on data discussed in this review.**Several drugs are approved for use in youth ≥ 12 years of age and others in clinical trials.

Below is a summary of clinical practices based on both human and animal studies regarding the metabolic programming of obesity, to reduce the long-term risk of obesity in children. Many of these have been recommended by professional organizations including the International Federation of Gynecology and Obstetrics (FIGO), American College of Obstetricians and Gynecologists (ACOG), American Academy of Pediatrics (AAP) and National Institutes of Health (NIH) (152). **Figure 4** incorporates these recommendations and reviews current therapeutic strategies to target obesity, as well as epigenetic modifiers and potential pathways to target based on preclinical and clinical studies discussed in this review, both for the prevention and treatment of childhood obesity.

## Conclusion

Here, we have summarized the mechanistic underpinnings of the metabolic programming of obesity, as well as the epigenetic pathways that “program” obesity, which should be considered as potential therapeutic targets both for the prevention and treatment of childhood obesity. We hope that this review challenges the commonly held belief that obesity is the result of a lack of motivation or conviction to practice healthy lifestyle choices, both in the individual and as a community. Several factors resist changes in feeding behavior and adiposity, some of which have been described in this review. We hope that further understanding of the metabolic conditioning of obesity both in the clinical and research arenas will help us collectively address the global epidemic of obesity.

## Author contributions

AR, CL, and VT conceived the manuscript and undertook research. AR wrote the first draft of the manuscript. VVT is responsible for the integrity of the content. All authors contributed to the article and approved the submitted version.

## Funding

Research reported in this publication was supported by National Institute of Diabetes and Digestive and Kidney DIseases of the National Institutes of Health under award number: K23DK110539 and P30DK026687. The content is solely the responsibility of the authors and does not necessarily represent the official views of the National Institutes of Health.

## Acknowledgments

The authors would like to thank Drs. Alicja A. Skowronski and Rudy Leibel for the helpful discussions about metabolic programming. The figures were created with BioRender.com.

## Conflict of interest

The authors declare that the research was conducted in the absence of any commercial or financial relationships that could be construed as a potential conflict of interest.

## Publisher’s note

All claims expressed in this article are solely those of the authors and do not necessarily represent those of their affiliated organizations, or those of the publisher, the editors and the reviewers. Any product that may be evaluated in this article, or claim that may be made by its manufacturer, is not guaranteed or endorsed by the publisher.
